# Bilirubin as a prognostic marker in patients with pulmonary arterial hypertension

**DOI:** 10.1186/1471-2466-10-22

**Published:** 2010-04-22

**Authors:** Yasuko Takeda, Yutaka Takeda, Shigehiro Tomimoto, Tomomitsu Tani, Hitomi Narita, Genjiro Kimura

**Affiliations:** 1 Department of Cardio-renal Medicine and Hypertension, Nagoya City University Graduate School of Medical Sciences, Nagoya, Aichi, Japan; 2 Division of Cardiology, Nagoya City Rehabilitation Center, Nagoya, Aichi, Japan

## Abstract

**Background:**

Liver dysfunction reflects the status of heart failure, with congestion and low perfusion of the liver serving as causative mechanisms. Previous studies demonstrated relationship between the results of liver function test and the prognosis in patients with heart failure. However, few studies have examined this relationship in patients with pulmonary arterial hypertension (PAH).

**Methods:**

The subjects were 37 patients with PAH (8 men and 29 women; 18 with idiopathic PAH and 19 with connective tissue disease-associated PAH). A blood test was performed after a 3-month period free from hospitalization and without changes in functional class, treatment, heart sounds, body weight, or heart rate.

**Results:**

In a mean follow-up period of 635 ± 510 days, 12 patients died due to heart failure, 2 died due to pulmonary hemorrhage, and 23 patients survived. Cox proportional hazard analyses identified functional class (p < 0.001), plasma concentration of brain natriuretic peptide (BNP) (p = 0.001), and hyperbilirubinemia (serum total bilirubin > 1.2 mg/dL; p < 0.001; hazard ratio = 13.31) as predictors of mortality. Patients with hyperbilirubinemia had a worse functional class (P = 0.003), a higher right atrial pressure (p < 0.001), a higher plasma concentration of BNP (p = 0.004), and a larger Doppler right ventricular index of the right ventricle (p = 0.041).

**Conclusion:**

Elevated serum bilirubin is a risk factor for death in patients with PAH.

## Background

Patients with pulmonary arterial hypertension (PAH) have an increased risk of mortality, and half of the patients in World Health Organization (WHO) functional class IV die within 3 years, even after treatment with epoprostenol [1]. Right heart failure is a major cause of death in patients with PAH [1]. The prognosis of PAH is predicted by markers of heart failure including WHO functional class [1], the plasma concentration of brain natriuretic peptide (BNP) [2], history of right heart failure, 6-minute-walk distance, mean blood pressure of the right atrium [1], and Doppler right ventricular index (Tei index of the right ventricle)[3]. Despite use of these markers, however, prediction of survival for each patient remains as a challenge in cardiology.

Liver dysfunction reflects the status of heart failure, with congestion and low perfusion of the liver serving as causative mechanisms [4-7]. The prognosis of heart failure has been associated with serum levels of aspartate aminotransferase and total bilirubin [4], but few studies have examined data from liver function tests as potential prognostic markers for patients with PAH. Therefore, we sought to determine whether the results of liver function tests could be used to predict prognosis in such patients.

## Methods

### Enrollment of patients

From August 2005 to March 2009, 62 consecutive patients with PAH of functional class II to IV were referred to our hospital to receive further care. Diagnosis of PAH was based on the results of right heart catheterization at rest [8]; mean pulmonary artery pressure > 25 mm Hg, pulmonary capillary wedge pressure ≤ 15 mm Hg, and pulmonary vascular resistance > 3 Wood units. Pulmonary hypertension with left heart valve disease, left ventricular ejection fraction < 50% (Venice Group 2), chronic obstructive pulmonary disease, interstitial lung disease, lung parenchymal disease, sleep disordered breathing, chronic exposure to high altitude, or developmental abnormalities (Venice Group 3), or thromboembolism in a lung perfusion scan (Venice Group 4) were not considered as PAH. Only patients with idiopathic PAH (Venice Group 1.1) or PAH associated with connective tissue disorder (CTD) (Venice Group 1.3.1) [9] were enrolled in the study.

One patient with Venice Group 1.2 (familial) PAH, 21 patients with congenital systemic-to-pulmonary shunt (Venice Group 1.3.2), 3 with parenchymal liver disease (Venice Group 1.3.3), and 1 with adult immune deficiency syndrome (Venice Group 1.3.4) were excluded from the study. We did not encounter patients with drug-induced PAH (Venice Group 1.3.5), thyroid disease, glycogen storage disease, Gaucher's disease, hereditary hemorrhagic telangiectasia, hemoglobinopathies, myeloproliferative disorders, or splenectomy (Venice Group 1.3.6). No patients had PAH in Venice Group 1.4 (pulmonary veno-occlusive disease or pulmonary capillary hemangiomatosis) proven by pathological examination or specific medical history including an abnormal response to pulmonary vasodilators. We also did not encounter any patient with PAH in Venice Group 1.5 (persistent pulmonary hypertension of the newborn).

Thirty-seven patients (8 men and 29 women) participated in the study after giving informed consent. These patients received a baseline examination after a 3-month period free from hospitalization for any cause and without changes in WHO functional class, treatment, heart sounds, body weight (> 2.0 kg), or heart rate (> 20 beat/min). Our protocol permitted body-weight-targeted adjustment of dose of diuretics. The baseline was defined as the day on which a blood examination was performed. The institutional ethics committee approved the study protocol.

### Baseline examinations

Blood samples for liver function tests were drawn from a peripheral vein after an overnight fast. For measurement of the plasma concentration of BNP, samples were immediately placed on ice and centrifuged at 4°C. BNP was measured using an immunoradiometric assay by an external laboratory (SRL Inc., Tokyo, Japan). Aspartate aminotransferase, alanine aminotransferase, lactate dehydrogenase, and total bilirubin were measured in the core laboratory at our hospital. The normal ranges are 13-38, 6-27, 119-229, and 0.3-1.2 mg/dL, respectively. Hyperbilirubinemia was defined as serum total bilirubin > 1.2 mg/dL.

A trained sonographer recorded all echocardiograms using a commercially available system (SSA-770A, Toshiba Medical Co., Tokyo, Japan). Cardiac index was calculated using a Doppler echo technique [10] and right ventricular function was determined from the Doppler right ventricular index; the definition and measurement procedure have been described elsewhere [11]. The pressure gradient between the right ventricle and right atrium was calculated with the simplified Bernoulli equation [11]. The sweep speed was 150 mm/s. All measurements were made at the end of expiration.

### Right heart catheterization

Right heart catheterization was performed in 26 patients within 14 days before or after the day of the blood test, without any change of treatment in the intervening period. The mean right atrial pressure and mean pulmonary arterial pressure obtained in this procedure were used for survival analysis. All the patients in the study underwent catheterization, but we did not use hemodynamic data for survival analysis in 11 patients due to a change of treatment between catheterization and the blood test.

### Statistical analysis

Statistical analyses were performed with the Statistical Package for Social Science version 15.0 for Windows (SPSS Inc., Chicago, IL). Survival times were defined as the time from baseline until July 31, 2009. All data but the concentrations of BNP expressed as means ± standard deviations. The concentrations of BNP were transformed to their natural logarithms to normalize the distribution for testing differences. The BNP data are expressed as the median with 25th and 75th percentiles. Comparisons of parameters between two groups were made by unpaired Student t-test or Mann-Whitney U test, as appropriate, and comparison among three groups was made by one-way analysis of variance with a Scheffe post hoc test. Categorical variables were compared with a Fisher exact test in the case of a 2 × 2 contingency table and a chi-square test for a 3 × 2 contingency table. The prognostic values of the variables were tested in univariate Cox proportional-hazards regression analyses. The results are expressed as hazard ratios with 95% confidence intervals. Survival curves were derived using the Kaplan-Meier method. A value of p < 0.05 was considered statistically significant in all analyses.

## Results

### Characteristics of the patients

As shown in Table 1, 18 patients had idiopathic PAH and 19 had CTD-associated PAH. All patients in WHO functional class III or IV and 11 patients in class II received oxygen therapy. The follow-up period was 635 ± 510 days and no patients dropped out of follow-up until completion of the analysis on July 31, 2009. During the study period, 12 patients died of heart failure, 2 died due to pulmonary hemorrhage, and 23 survived until the end of the study. No patients received lung or heart-lung transplantation, despite some being listed for lung transplantation. Death occurred in 9 patients in WHO functional class IV at baseline (90% of the patients in this class), 3 in class III (25%), and 2 (13%) in class II (p < 0.001). Treatment failure occurred in 9 patients in class IV (90%), 5 in class III (42%), and 6 in class II (40%), where treatment failure was defined as death, hospitalization due to a cardiovascular event including syncope, escalation of pulmonary vasodilator therapy, use of inotropic agents, or worsening of WHO functional class (p = 0.028).

**Table 1:** Characteristics of the patients

Variables	All patients	Hyper- bilirubinemia	Normo- bilirubinemia	p Value
	n = 37	n = 6	n = 31	
Age, yrs	49 ± 18	53 ± 15	48 ± 19	0.62
Women	29 (78)	1	7	0.75
Height, cm	153 ± 20	152 ± 7	153 ± 19	0.36
Body weight, kg	50 ± 10	43 ± 10	51 ± 9	0.04
Systolic blood pressure, mm Hg	113 ± 20	104 ± 12	114 ± 22	0.26
Diastolic blood pressure, mm Hg	70 ± 14	61 ± 11	71 ± 14	0.10
Heart rate, beats per minute	88 ± 14	95 ± 8	86 ± 15	0.18
WHO Functional class II/III/IV	15/12/10 (41/32/27)	1/0/5	14/12/5	0.003
Idiopathic/CTD-associated	18/19 (49/51)	4/2	14/17	0.34
Treatment at baseline				
Epoprostenol alone	11 (30)	4	7	
Sildenafil alone	4 (11)	0	4	
Bosentan alone	6 (16)	0	6	
Beraprost alone	13 (35)	2	11	
Epoprostenol + Sildenafil	1 (3)	0	1	
Sildenafil + Beraprost	1 (3)	0	1	
Sildenafil + Bosentan + Beraprost	1 (3)	0	1	
Treatment at the end of follow-up				
Epoprostenol alone	17 (46)	5	12	
Sildenafil alone	5 (14)	0	5	
Bosentan alone	5 (14)	0	5	
Beraprost alone	6 (16)	0	6	
Epoprostenol + Sildenafil	3 (8)	1	2	
Sildenafil + Beraprost	1 (3)	0	1	
Mean right atrial pressure, mm Hg*	8 ± 4	14 ± 2	7 ± 3	<0.001
Mean pulmonary arterial pressure, mm Hg*	52 ± 20	71 ± 4	50 ± 19	0.002
Cardiac index, l/min/m^2^	2.87 ± 0.80	2.61 ± 0.90	2.90 ± 0.81	0.46
Doppler RV index	0.64 ± 0.30	0.86 ± 0.23	0.58 ± 0.29	0.04
Plasma BNP, pg/ml^†^	170 [70, 796]	1062 [833, 1210]	337 [141, 959]	0.003
AST, U/L	34 ± 38	58 ± 87	29 ± 18	0.46
ALT, U/L	36 ± 55	67 ± 129	29 ± 23	0.51
LDH, U/L	247 ± 82	315 ± 167	234 ± 48	0.29
Bilirubin, mg/dL	0.9 ± 0.9	2.4 ± 1.5	0.6 ± 0.3	N/A
Uric acid, mg/dL	6.8 ± 2.5	7.6 ± 4.7	6.6 ± 1.8	0.64

Values are shown as a number (%) or as a mean ± SD as appropriate; ALT = alanine aminotransferase, AST = aspartate aminotransferase, BNP = brain natriuretic peptide; CTD = connective tissue disorder; LDH = lactate dehydrogenase, PAH = pulmonary arterial hypertension, RV = right ventricle, WHO = World Health Organization. *n = 26, ^† ^Data are given as a median [25th, 75th percentile].

Plasma concentration of BNP differed among the patients in each WHO functional class. The median value (25th percentile, 75th percentile) of the patients in class II, III, and IV were 53 (25, 128) pg/mL, 309 (148, 502) pg/mL, and 932 (806, 1210) pg/mL, respectively. The differences between each pair of the classes were significant: p < 0.001 between class II and III, p = 0.002 between class III and IV, and p < 0.001 between class II and IV, respectively.

### Mortality predictors

**Figure 1:**
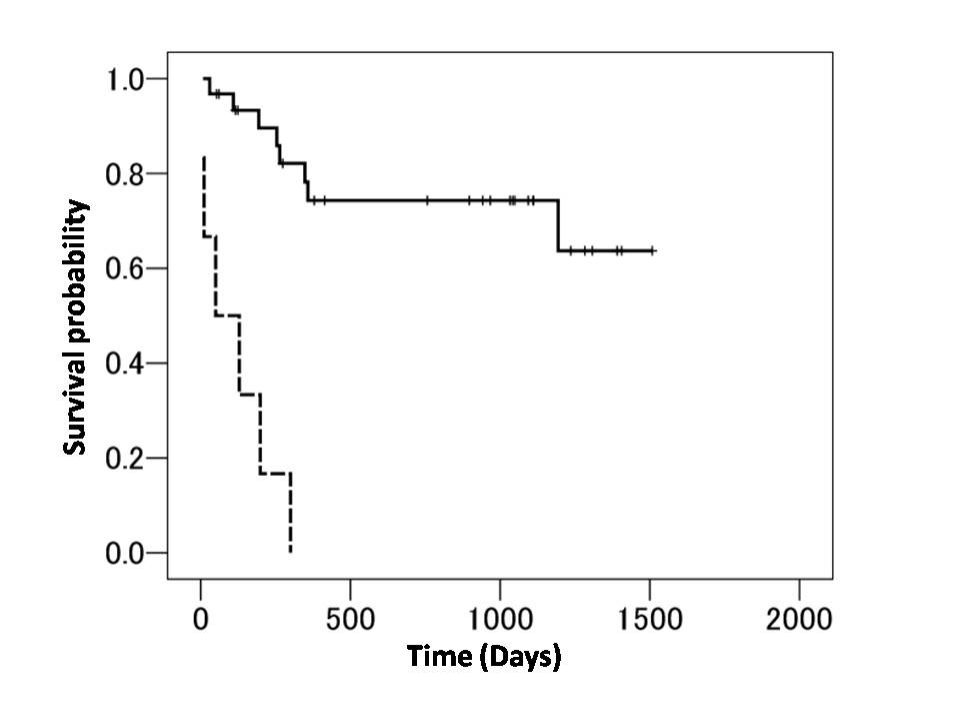
**Kaplan-Meier estimates of survival in patients with or without hyperbilirubinemia**. The broken and continuous lines indicate the patients with and without hyperbilirubinemia, respectively. Hyperbilirubinemia was defined as serum total bilirubin > 1.2 mg/dL.

Univariate Cox proportional hazard analyses were performed using data from liver function tests and conventional mortality predictors: mean right atrial pressure, mean pulmonary arterial pressure, cardiac index, WHO functional class, and plasma concentration of BNP. These analyses demonstrated that WHO functional class (p < 0.001), BNP (p = 0.001), the serum concentration of bilirubin (p = 0.001), and hyperbilirubinemia (p < 0.001) predicted the risk of death. Patients with hyperbilirubinemia had markedly worse survival than those with normobilirubinemia (Figure 1), and had a worse WHO functional class (P = 0.003), higher mRAP (p < 0.001), higher BNP (p = 0.004), and a larger Doppler RV index (p = 0.041). The mean pulmonary arterial pressure did not differ significantly between patients with hyperbilirubinemia and those with normobilirubinemia (Table 1).

Given the interdependence between WHO functional class and plasma BNP concentration, two models of bivariate analysis were analyzed: a model using WHO functional class and hyperbilirubinemia (both categorical data, Model 1) and a model using BNP and bilirubin concentration (both continuous data, Model 2). In these models, hyperbilirubinemia and bilirubin concentration were found to be predictors of a risk of death independently of WHO functional class and BNP, respectively (Table 2).

**Table 2:** Results of Cox proportional hazard analyses

	Univariate		Model 1^†^		Model 2^‡^	
Variables	p	Hazard ratio (95% CI)	p	Hazard ratio (95% CI)	p	Hazard ratio (95% CI)
Age, year	0.51	1.01 (0.98--1.04)				
Women	0.71	0.78 (0.22--2.83)				
CTD-associated	0.29	0.49 (0.16--1.46)				
Mean RA pressure, mm Hg*	0.05	1.19 (1.00--1.43)				
Mean PA pressure, mm Hg*	0.34	0.99 (0.95--1.02)				
Cardiac index, L/min/m^2^	0.70	1.14 (0.58--2.24)				
WHO functional class	<0.001	4.95 (2.17--11.32)	0.009	3.30 (1.35--8.03)		
Doppler RV index	0.17	3.03 (0.61--14.99)				
BNP, lognormal pg/dL	0.001	2.79 (1.55--5.04)			0.004	2.35 (1.31 -- 4.21)
Elevated AST	0.34	0.37 (0.05--2.84)				
Elevated ALT	0.30	0.34 (0.04--2.60)				
Elevated LDH	0.17	2.10 (0.73--6.12)				
Hyperbilirubinemia	<0.001	13.31 (3.91--45.30)	0.02	4.81 (1.24--18.73)		
Bilirubin, mg/dL	0.001	3.64 (1.73--7.68)			0.02	2.28 (1.15 -- 4.52)

BNP = brain natriuretic peptide; CTD = connective tissue disorder; PA = pulmonary artery, RV = right ventricle, WHO = World Health Organization. *n = 26, ^† ^Bivariate model using WHO functional class and hyperbilirubinemia, ^‡ ^Bivariate model using BNP and bilirubin concentration.

## Discussion

The current study suggests that hyperbilirubinemia is associated with advanced right heart failure and reduced survival in patients with PAH, compared with patients with a normal serum bilirubin concentration. Hyperbilirubinemia was associated with a worse WHO functional class, higher right atrial pressure, higher BNP, and a larger Doppler right ventricular index. Thus, our results suggest that hyperbilirubinemia is a powerful, yet simple and readily available, marker of advanced right heart failure and poor prognosis in patients with PAH.

Our results also show that bilirubin is more sensitive to hemodynamic abnormality than transaminases. This is consistent with a recent investigation showing the superiority of bilirubin over transaminases in sensitivity to hemodynamic abnormality in patients with left heart failure [12]. This study also showed that the serum bilirubin level is a strong, independent predictor of an adverse prognosis in patients with left heart failure, even after adjustment for a variety of demographic, clinical, and biochemical variables [12]. Serum bilirubin has also been shown to correlate with hemodynamic parameters such as right atrial pressure [6,13] and severity of tricuspid regurgitation [6,13], and biliary obstruction may potentially be caused by high hepatic venous pressure [14,15]. Conversely, correlations of transaminases with these parameters have not been found [5,14].

There are several limitations to our study. The sample size was small and further studies in a larger number of patients are required to confirm the conclusions. Doppler echocardiography was used to measure cardiac index because thermodilution or Fick's method was unavailable due to severe tricuspid regurgitation or use of oxygen therapy, respectively. We did not use the 6-minute-walk test in the statistical analyses because many of our patients could not tolerate this stress, and performance of a precise walk stress test is difficult in patients receiving continuous oxygenation therapy. The number of patients with CTD-associated PAH and the high mortality rate might reflect a referral bias because our institute is an academic center for cases of pulmonary hypertension that have treatment difficulties. Finally, normal serum concentration of bilirubin does not guarantee that the risk of death is always low.

## Conclusions

In conclusion, the study demonstrated that hyperbilirubinemia is associated with advanced right heart failure and markedly reduced survival in patients with PAH. Therefore, serum bilirubin should be monitored in management of patients with PAH.

## Competing interests

The authors declare that they have no competing interests.

## Authors' contributions

All the authors joined to design of this study and to write this manuscript. Yasuko T analyzed patients' data especially serum concentration of bilirubin. Yutaka T gave original concept of this study. ST and TT worked for analysis of survival data. HN performed statistical analyses. GK watched the safety of this study.

## Pre-publication history

The pre-publication history for this paper can be accessed here:

http://www.biomedcentral.com/1471-2466/10/22/prepub
